# Expression of transglutaminase-2 (TGM2) in the prognosis of female invasive breast cancer

**DOI:** 10.1038/s44276-023-00030-w

**Published:** 2024-01-24

**Authors:** Fiona M. Blows, H. Raza Ali, Wei Cope, Paul D. P. Pharoah, Claire V. S. Pike, Elena Provenzano, Peter Coussons

**Affiliations:** 1 Department of Oncology, University of Cambridge, Cambridge, UK; 2 Biomedical Research Group, Department of Biomedical and Forensic Sciences, Faculty of Science and Technology, Anglia Ruskin University, Cambridge, CB1 1PT UK; 3 Cancer Research UK Cambridge Institute, University of Cambridge, Li Ka Shing Centre, Robinson Way, Cambridge, CB2 0RE UK; 4 Department of Computational Biomedicine, Cedars Sinai Medical Center, Los Angeles, CA USA; 5 Cambridge Breast Unit, Addenbrooke’s Hospital, Cambridge University Hospital NHS Foundation Trust and NIHR Cambridge Biomedical Research Centre, Cambridge, CB2 2QQ UK

## Abstract

**Background:**

Transglutaminase 2 (TGM2) is a protein expressed in several isoforms in both intra- and extra-cellular tissue compartments. It has multiple functions that are important in cancer biology and several small studies have suggested expression of TGM2 in breast cancers is associated with a poorer prognosis. The aim of this study was to evaluate the role of intra-cellular and extra-cellular TGM2 expression in breast cancer and to determine whether there were any differences by hormone receptor status.

**Methods:**

We carried out TGM2 immunostaining of tissue micro-arrays comprising 2169 tumour cores and scored these for both intra- and extra-cellular and expression.

**Results:**

Intra-cellular (tumour cell) TGM2 positivity was associated with a better prognosis (HR = 0.74, 95% CI 0.59–0.92) with a larger effect stronger in hormone-receptor-negative cases (HR = 0.56, 95% CI 0.37–0.85). Extra-cellular (stromal) TGM2 expression was associated with a poorer prognosis (HR = 1.47, 95% CI 1.06–2.03) with a stronger association in hormone-receptor-positive cases (HR = 1.60, 95% CI 1.09–2.34).

**Conclusion:**

Tissue compartment and hormone receptor status differences in the effect of TGM2 expression on clinical outcomes of breast cancer may reflect the different functions of TGM2.

## Background

Breast cancer is a heterogeneous disease, and the prognosis of early breast cancer varies substantially depending on clinico-pathological features and molecular characteristics. These factors are used to guide treatment decisions. The PREDICT breast prognostication and treatment benefit model is based on age at diagnosis, mode of detection, tumour size, tumour grade, number of positive lymph nodes and expression of estrogen receptor (ER), progesterone receptor (PR), human epidermal growth factor receptor 2 (HER2) and the marker of proliferation Kiel 67 (KI67) [1, 2]. Many other tumour proteins have been evaluated as prognostic biomarkers. For example, cytokeratin 5/6 (CK5/6) and epidermal growth factor receptor (EGFR) are markers of disease that originated from basal cells [3].

Transglutaminase 2 (TGM2), a member of a family of calcium-dependent transglutaminase enzymes, is a multifunctional protein, under the control of intracellular GTP and calcium levels. TGM2 modifies proteins by catalysing the formation of ε(γ-glutamyl)-lysine bonds; it is instrumental in protein polymerisation and incorporation of diamines and polyamines into proteins [4]. This multiply-spliced gene product of *TGM2* is expressed both intra- and extracellularly and its diverse roles are conformation and isoform dependent [5]. TGM2 is expressed in many tissues, but it not expressed in normal breast epithelium [6]. However, *TGM2* and TGM2 are up-regulated in breast cancer [6] and TGM2 has been shown to play multiple roles in the biology of breast cancer including cell adhesion, invasion and survival [7], programmed cell death [8], cell signalling [9], and metabolic reprogramming [10]. TGM2 has also been suggested to limit metastasis of colon cancer cells by effectively forming restrictive fibrillar networks in the stroma [11]. The two major isoforms of TGM2, TGM2-long and the truncated TGM2-short, are proposed to have opposing roles in cancer, being pro-cell-survival and pro-apoptotic, respectively [12].

Thus, tumour TGM2 expression is a good candidate as a biomarker of prognosis in breast cancer. Several studies have reported on the association between TGM2 expression and prognosis in breast cancer [6, 13, 14] with increased expression being associated with a poorer prognosis. However, the largest of these studies [13] was based on just 412 patients and no study has evaluated the role of TGM2 expression according to tumour hormone receptor status or has evaluated both intra-cellular expression and extra-cellular expression. The aim of this work was to assess the association between expression of TGM2 in both tumour and stromal compartment in invasive hormone receptor positive and hormone receptor negative female breast cancer in a cohort of 2169 women with early invasive breast cancer.

## Materials and methods

### Study population

The Study of Epidemiology and Risk in Cancer Heredity (SEARCH), is population-based and consists of two study populations. The populations were prevalent cases (diagnosed at under 55 years of age from 1991 to 1996 and still alive when the study started in 1996), and incident cases (diagnosed at under 70 years of age after 1996). Cases were identified through the East Anglia Cancer Registry until 2003 and subsequently through the Eastern Cancer Registry and Information Service (ECRIC). Follow-up by the Cancer Registry was carried out through national death registration. Participants with breast cancer (ICD10 code C50) recorded on Part I of the death certificate were considered to have died from breast cancer. SEARCH is approved by the National Research Ethics Service Cambridgeshire Committee and all participants provided written, informed consent.

### Tissue microarray (TMA) construction

Pathology blocks from 2169 patients were retrieved for tissue micro-array construction. Tissue microarray blocks were constructed using donor pathology blocks taken at the time of primary surgery before any treatment. The position for core selection was guided by slides stained with haematoxylin and eosin with areas of invasive carcinoma marked by a pathologist. Each tumour is represented by a single 0.6 mm core in a TMA constructed from paraffin-embedded tissue blocks.

### Assigning hormone-receptor-status to cases

Sections from each TMA were previously stained for ER and PR and scored using the Allred system [15]. Allred scores of three or more were considered positive. ER status data from the clinical record were also available for some cases with missing data from the TMAs. Cases were designated hormone-receptor-positive if either oestrogen receptor (ER) or progesterone receptor (PR) status was positive. Where data were missing for one of the markers, as was the case in 65 cases (2 lacked ER status and 63 lacked PR status), hormone-receptor status was based on the single marker – some of the hormone-receptor-negative cases may thus be misclassified. However, where both ER and PR status was known there was agreement in 84% of cases, thus misclassification is suggested to have happened only in approximately 10 cases.

### IHC staining of TGM2

Antigen retrieval was performed using an antigen access unit, supplied by Menarini Diagnostics, with Menarini access tris buffer. The slides were transferred to an IntelliPATH autostainer; all of the incubations were at ambient temperature and the following reagents and timings were applied: five minutes with Menarini peroxidase block; five minutes with Menarini casein block; one hour with Abcam TGM2 antibody (ab2386) diluted to a concentration of 1:500; ten minutes with Menarini universal probe; and fifteen minutes with horseradish peroxidase. These steps were all followed by one wash with Menarini buffer 1, a ten-minute incubation with diaminobenzidine (DAB), two washes with water, one minute with Mayers haematoxylin, and finally one wash with water.

When present in tumour cells, TGM2 expression was almost exclusively in the cytoplasm. The tissue sections had the expected features of breast tumour tissue, including blood vessels, ducts, stroma and fibroblasts. Where cytoplasmic staining of TGM2 occurred, it was generally present in some, but not all, of the tumour cells in the sample. Where there was a mixture of stained and unstained cells, there were often small clusters of stained cells; in general, they were moderately well-distributed throughout the section.

### Scoring TGM2 expression

**Fig. 1:**
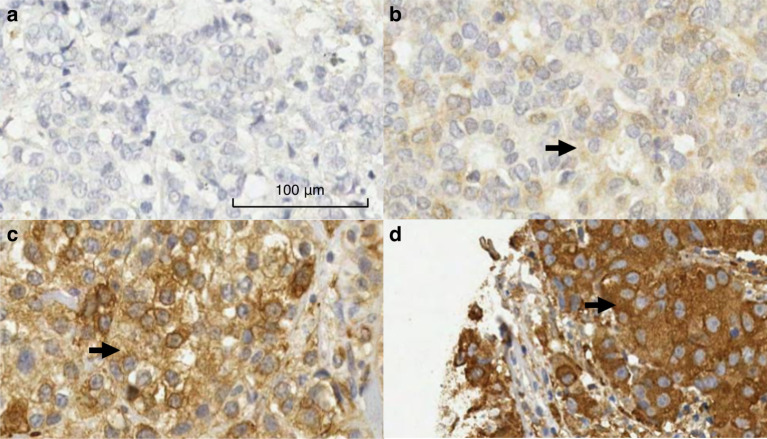
Representative images of the immunohistochemical staining of intra-cellular TGM2. **a** No staining (score of 0). **b** No staining, weak staining (representative score of 1 indicated with a green arrow) and medium staining. **c** Mainly medium (representative score of 2 indicated with a green arrow) staining with a few areas of strong staining. **d** Strong staining (representative score of 3 indicated with a black arrow).

**Fig. 2:**
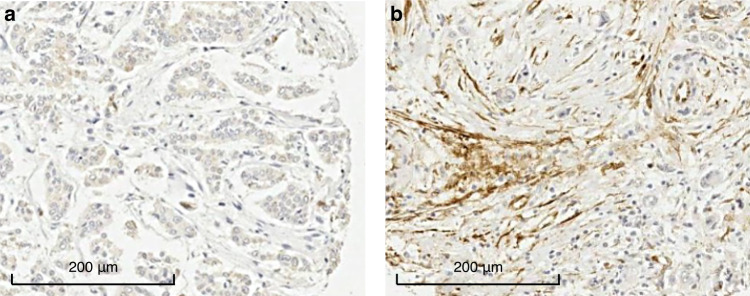
Representative images of the immunohistochemical staining of breast tumour-stromal tissue for TGM2. **a** Extracellular TGM2 expression negative. **b** Extracellular TGM2 expression positive.

Intra-cellular TGM2 expression in tumour cells and extra-cellular expression in stromal tissue was scored by a pathologist (EP) and a trained technician and PhD candidate (FMB). TGM2 staining intensity was scored on a four-point scale (0 = no staining, 1 = weak staining, 2 = medium staining and 3 = strong staining) and the percentage of tumour cells positive was estimated to the nearest ten per cent. Only invasive tumour cells were scored. Where the intensity was not consistent the most prevalent intensity was used. Scores of 10% or more were considered positive (Fig. 1). TGM2 was generally evenly distributed when present in the stroma; cases with medium or strong staining of 10% or more of the stroma were considered positive (Fig. 2).

### Statistical analyses

All analyses were performed using R Statistical Software v4.1.2 [16], implemented in R studio v2022.7.1.554 [17]. Packages used were *gap* [18], *tidyverse* [19], *survival* [20], *survminer* [21] and *tableone* [22]. Associations between TGM2 expression and categorical variables were evaluated using standard χ^2^ tests. Associations between TGM2 expression and death from breast cancer were investigated using Cox proportional hazards regression. Follow-up time was taken from the date of diagnosis. As participants were recruited after diagnosis (left truncation), time at risk was taken from the date of entry into the study. Follow-up was censored on the date of death or on the date last known to be alive or at 15 years after diagnosis, whichever came first. The proportional hazards assumption was tested by assessing the correlation of the weighted Schoenfeld residuals with time. Analyses were carried out for all cases and for hormone-receptor-positive and hormone-receptor-negative cases separately.

## Results

Tumours from 2169 cases were stained for TGM2. The numbers of cases by intra-cellular (tumour cell) TGM2 expression status and age group, hormone-receptor-status, grade, stage and extra-cellular (stromal) TGM2 expression are shown in Table 1. Intracellular and extracellular TGM2 status were only weakly correlated (Kappa = 0.095, *P* = 0.003). Outcome data were not available for 13 cases and so 2156 cases were included in the final set for time-to-event analyses. Of these, 431 (17%) died of breast cancer within 15 years of diagnosis. The fully adjusted models were based on a complete case analysis *n* = 1925 with complete data).

**Table 1:** Characteristics of cases by tumour intra-cellular TGM2 expression status.

	TG2-status		*p*-value
	Negative	Positive	
	*n* (%)	*n* (%)	
Total	1380 (64)	789 (36)	
Age group			
20–30	5 (0.4)	7 (0.9)	0.07
30–<40	99 (7.2)	61 (7.7)	
40–<50	385 (27.9)	253 (32.1)	
50–<60	542 (39.3)	295 (37.4)	
≥60	349 (25.3)	173 (21.9)	
Hormone receptor status			
Negative	288 (21.2)	157 (20.1)	0.59
Positive	1070 (78.8)	623 (79.9)	
Missing	22	9	
Tumour grade			
1	242 (19.5)	110 (15.5)	0.01
2	552 (44.4)	361 (50.9)	
3	450 (36.2)	238 (33.6)	
Missing	136	80	
Stage			
1	598 (44.0)	345 (45.2)	0.06
2	712 (52.4)	392 (51.4)	
3	42 (3.1)	15 (2.0)	
4	7 (0.5)	11 (1.4)	
Missing	21	26	
Stromal TGM2 expression			
Negative	304 (22.0)	128 (16.2)	<0.01
Positive	285 (20.7)	184 (23.3)	
Missing	791	477	
Breast cancer death			
Negative	1072 (78.1)	653 (83.3)	<0.01
Positive	300 (21.9)	131 (16.7)	
Missing	8	5	

**Fig. 3:**
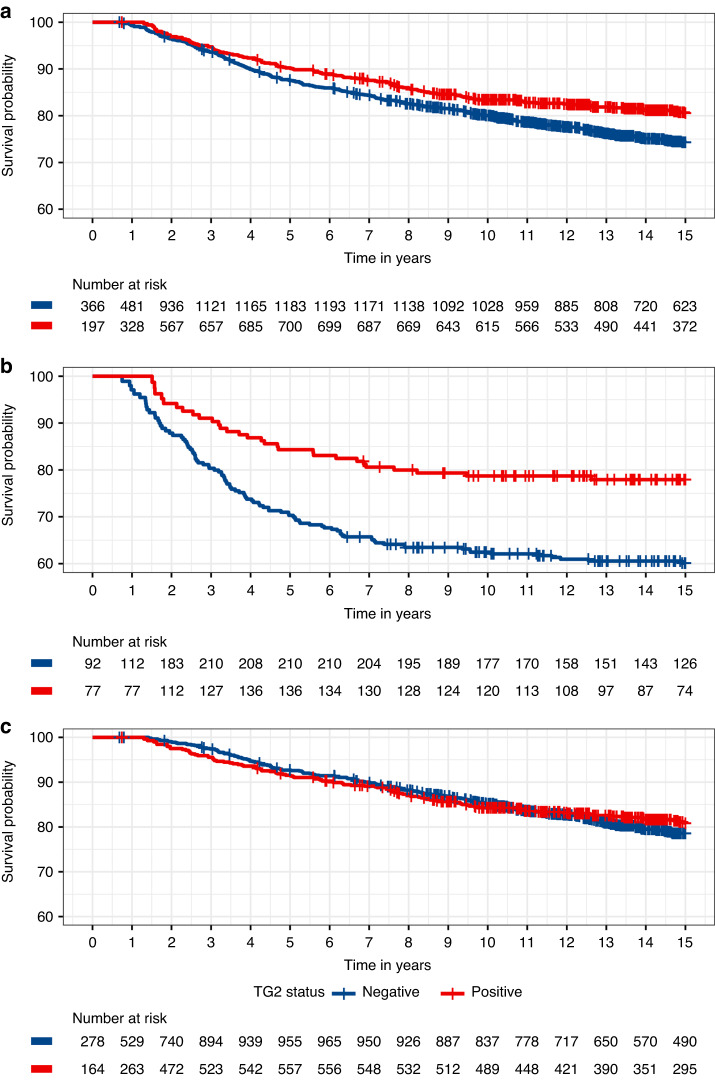
Kaplan–Meier cumulative survival (breast cancer-specific mortality) by intra-cellular TGM2 expression status. **a** All tumours. **b** Hormone-receptor-negative tumours. **c** Hormone-receptor-positive tumours.

In univariable analysis intra-cellular (tumour cell) TGM2 positivity was associated with a better prognosis (HR = 0.75, 95% CI 0.61–0.92; *P* = 0.0053). The proportional hazards assumption was not violated (*P* = 0.27). There was little difference in the hazard ratio estimate after adjusting for stage and grade, which are known to affect patient survival, (Fig. 3a, HR = 0.74, 95% CI 0.59–0.92; *P* = 0.0059). However, the effect was stronger in hormone-receptor-negative cases (Fig. 3b, HR = 0.56, 95% CI 0.37–0.85; *P* = 0.0064) than in hormone-receptor-positive cases (Fig. 3c, HR = 0.87, 95% CI 0.67–1.12; *P* = 0.28). This difference was nominally statistically significant (*P* = 0.037).

TGM2 expression data in the stroma was obtainable for 901 cases. TGM2 stromal expression was associated with a poorer prognosis in univariable analysis (HRadj = 1.60, 95% CI 1.19–2.14, *P* = 0.0018) with some attenuation after adjusting for stage and grade ((HR = 1.47, 95% CI 1.06–2.03, *P* = 0.02). The association was stronger in hormone-receptor-positive cases (HRadj = 1.60, 95% CI 1.09–2.34, *P* = 0.015) with little evidence of any effect in hormone-receptor-negative cases (HR = 0.92, 95% CI 0.49–1.71, *P* = 0.78). However, the difference was not statistically significant (*P* = 0.15). In hormone receptor positive disease the association the effect was bigger in the intra-cellular TGM2 expression negative cases (HRadj = 1.88, 95% CI 1.17–3.02, *P* = 0.0092) than intra-cellular TGM2 expression positive cases (HRadj = 1.10, 95% CI 0.57–2.09, *P* = 0.78). Again, this difference was not statistically significant (*P* = 0.23).

## Discussion

We have found modest evidence for an association between intra-cellular expression of TGM2 in the tumour cytoplasm and an improved prognosis in early breast cancer with a bigger effect in hormone-receptor-negative disease than in hormone-receptor positive disease. This is in contrast to previous studies that have evaluated the prognostic significance of TGM2 in breast tumours, all of which found that increased expression was associated with a poorer prognosis [6, 13, 14]. However, previous studies have not reported on the association stratified by hormone receptor status, nor did they report on the association with intra-cellular expression. One study reported only on the role of extra-cellular expression [14] and our findings support this observation as TGM2 expression in the stroma was associated with a poorer prognosis, an association that appeared to be restricted to hormone receptor positive disease. We used the Bayes False Discovery Probability (BDFP) to assist in the interpretation of each nominally significant association [23]. The BFDP is the probability that a reported significant association is a false positive given a specified prior probability that the alternative hypothesis is true. We have assumed that if the alternative hypothesis is true, the effect size is unlikely to be a hazard ratio greater than 1.5 or less than 0.67. As shown in Table 2, even under reasonably strong priors, the observed associations are quite likely to represent false positives. This is despite the large sample size of this study. Even larger sample sizes will be needed to confirm these associations with high confidence.

**Table 2:** Bayes False Discovery Probability (%) for observed significant findings (*P* < 0.05) given different prior probabilities of true association.

	Hazard ratio	*P*-value	Prior probability of true association			
			50%	20%	10%	5%
*T2G expression in tumour*						
All	0.75	0.0053	9	29	47	66
Hormone-receptor negative	0.56	0.0064	19	48	67	81
*TG2 expression in stroma*						
All	1.47	0.02	24	55	73	85
Hormone-receptor positive	1.60	0.015	23	55	73	85

The observed associations are intriguing in their specificity. They suggest that the function of TGM2 in breast tumours depends on hormone-receptor-status and differs depending on whether it is expressed inside or outside of the cell. The weak correlation between intra-cellular expression in tumour cells and extra-cellular expression in the stroma suggests that cells other than the tumour are the source of the extracellular protein. Indeed, TGM2 may be produced in and secreted from various cell types including the fibroblasts that occur in the tissue stroma [24]. It is plausible that the differences between in effects of intra- and extra-cellular expression reflect the many different functions of TGM2 [5]. For example, TGM2 has been shown to promote inflammatory signalling which would be expected to be associated with poorer outcomes [13] and targeted depletion of TGM2 inhibits metastasis, while overexpression of TG2 enhances metastasis [25]. On the other hand, tumour progression was increased and survival rate reduced in TGM2 knockout mice compared to wild-type [11] and TGM2 activation induces programmed cell death [8]. Additional complexity comes from the fact that the different isoforms may be differentially expressed in different tumours and have different functions. However, the antibody used for the present work detects total TGM2 and is unable to distinguish isoforms; isoform specific antibodies are not currently available.

A limitation of this study is the use of TGM2 expression derived from a single 0.6 mm core of tumour tissue. All tumours will have some degree of spatial heterogeneity in their morphology and molecular characteristics, and a single sample may not be representative of the tumour. Thus, the TGM2 expression based on a single core can be considered a form of random measurement error. Consequently, there will be some attenuation of any true associations.

In conclusion, we have found evidence to support previous publications that have suggested breast cancers that express TGM2 in the stoma are associated a poorer than those that do not express TGM2. A possible association of intra-cellular TGM2 expression with a better prognosis in invasive breast cancer has not been reported previously and needs confirmation in independent data sets.

## Data Availability

The data and analysis code (R script) to reproduce the results reported in this manuscript are available from the corresponding author on request.

## Supplementery information

Supplementary Fig. 1
Supplementary_data
