# Supplementary figures and images for: Expression of transglutaminase-2 (TGM2) in the prognosis of female invasive breast cancer
Source: BJC Rep. 2024 Jan 24;2:5. doi: 10.1038/s44276-023-00030-w (PMC11523935; doi:10.1038/s44276-023-00030-w)

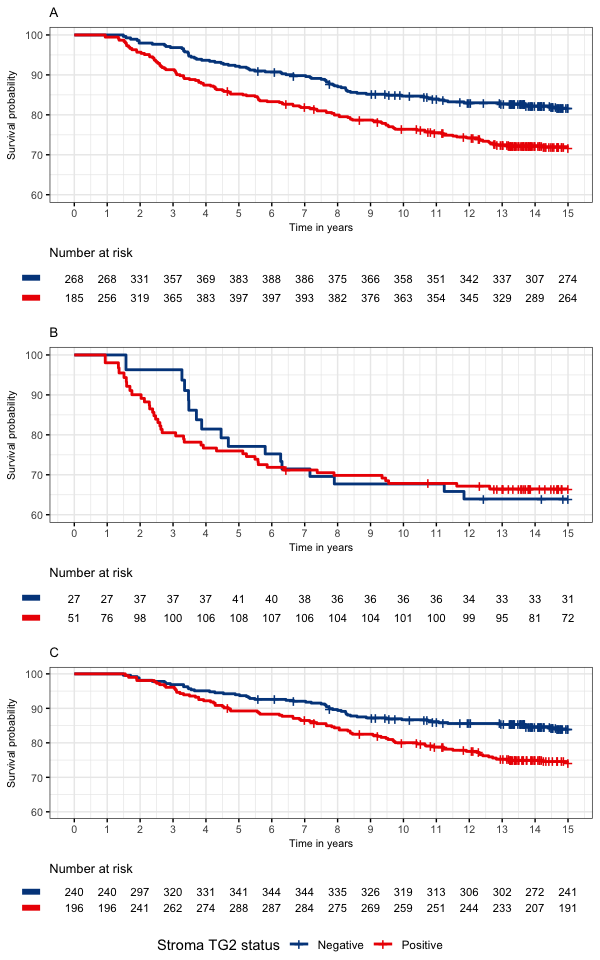

Supplement: Supplementary file 1 — Supplementary Fig. 1 [file 44276_2023_30_MOESM1_ESM.png]
